# Surgery for Idiopathic Scoliosis: Currently Applied Techniques

**DOI:** 10.4137/cmped.s2117

**Published:** 2009-03-04

**Authors:** Toru Maruyama, Katsushi Takeshita

**Affiliations:** 1 Department of Orthopaedic Surgery, Saitama Medical Center, Saitama Medical University Saitama, Japan.; 2 Department of Orthopaedic Surgery, Faculty of Medicine, The University of Tokyo Tokyo, Japan.

**Keywords:** scoliosis, surgery, instrumentation, fusion, fusionless

## Abstract

This review discusses the basic knowledge and recent innovation of surgical treatment for scoliosis. Surgical treatment for scoliosis is indicated, in general, for a curve exceeding 45 to 50 degrees by the Cobb’s method on the basis that:

1. Curves larger than 50 degrees progress even after skeletal maturity.
2. Curves larger than 60 degrees cause loss of pulmonary function, and much larger curves cause respiratory failure.
3. Greater the curve progression, the more difficult it is to treat with surgery.

Posterior fusion with instrumentation has been the standard form of surgical treatment for scoliosis. In modern instrumentation systems, more anchors are used to connect the rod and the spine, resulting in better correction and less frequent implant failures. Segmental pedicle screw constructs or hybrid constructs using pedicle screws, hooks, and wires are the trend of today. Anterior instrumentation surgery was once the choice of treatment for thoracolumbar and lumbar scoliosis because better correction could be obtained with shorter fusion levels. But in the recent times, superiority of anterior surgery for the thoracolumbar and lumbar scoliosis has been questioned. Initial enthusiasm for anterior instrumentation for the thoracic curve using video assisted thoracoscopy has faded out.

## Review

This review discusses the basic knowledge and recent innovation of surgical treatment for scoliosis. Since relatively little data are obtained regarding outcomes in the long-term or clinical outcomes such as patients’ satisfaction, the techniques are discussed mainly based on the radiological outcomes in the middle-term or sometimes based on short-term follow-up. 

## Indication of Surgery

Surgical treatment for scoliosis is indicated, in general, for a curve exceeding 45 to 50 degrees by the Cobb’s method on the basis that:

1. Curves larger than 50 degrees progress even after skeletal maturity. Thoracic curves with a magnitude between 50 and 75 degrees at skeletal maturity (Risser IV or V) progressed by an average of 29.4 degrees over the 40.5 years follow-up period.^1^ Curves greater than 55 degrees at skeletal maturity (partial or total fusion of the iliac apophyses) progressed by more than 0.5 degrees per year.^2^ Thoracic curves with an average Cobb angle of 60.5 degrees progressed to 84.5 degrees over the 50 year follow-up period.^3^
2. Curves larger than 60 degrees cause loss of pulmonary function, and much larger curves cause respiratory failure. In patients with curves between 60 and 100 degrees, total lung capacity was 68% of the predicted normal values.^4^ Nearly half of the patients with thoracic curve greater than 80 degrees had shortness of breath, by an average age of 42 years.^5^ Vital capacity below 45% of the normal value and a Cobb angle greater than 110 degrees were risk factors to develop respiratory failure and earlier death.^6^
3. Greater the curve progression, the more difficult it is to treat surgically, with more surgical anchors being necessary, duration of surgery prolonged, increased blood loss, and higher surgical complication rate.

Sometimes patient’s motivation to straighten his/her spine by surgery should be respected, especially for the patient with a gray zone curve and a Cobb angle of 40 to 45 degrees.

Surgical treatment for scoliosis can be divided into fusion surgery and fusionless surgery.

## Fusion Surgery

### Posterior instrumentation

**Figure 1:**
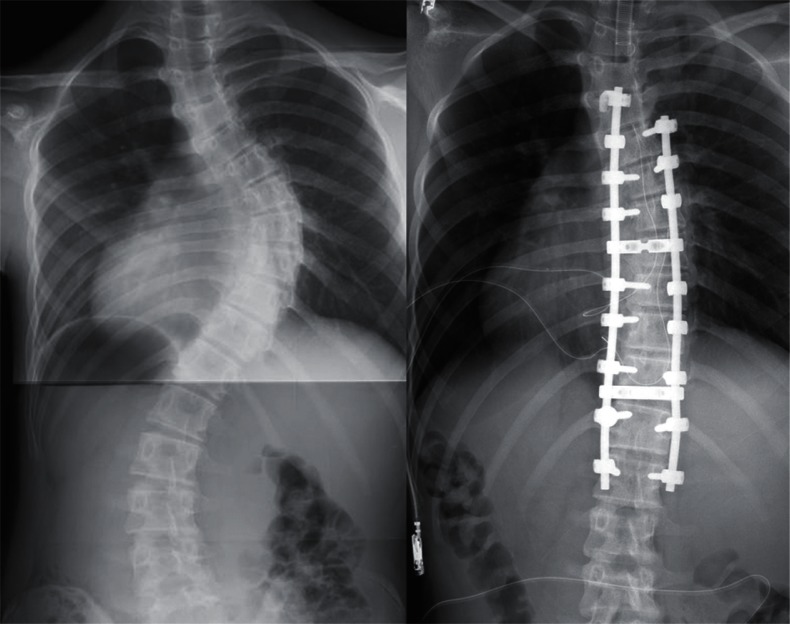
Segmental pedicle screw constructs. Right thoracic curve between the T5 and T11 was corrected from 68 to 25 degrees.

**Figure 2:**
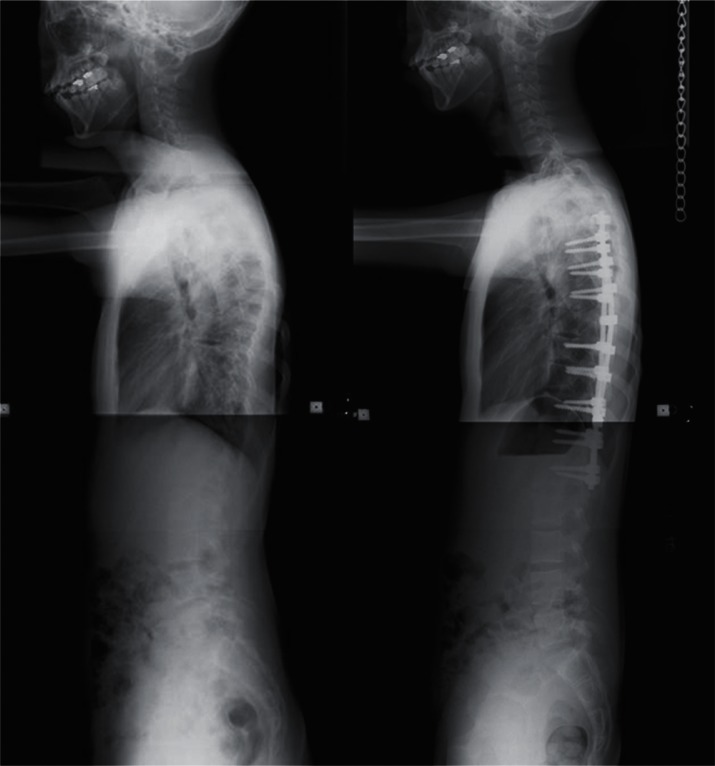
Segmental pedicle screw constructs. Lateral radiographs before and after surgery.

**Figure 3:**
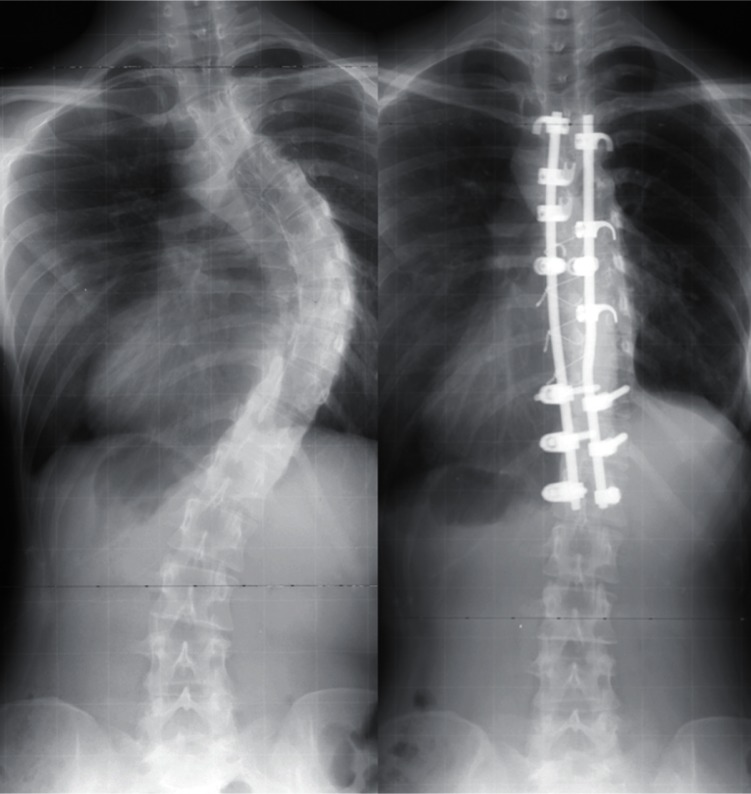
Hybrid constructs using pedicle screws, hooks, and wires. Right thoracic curve between the T5 and T11 was corrected from 70 to 23 degrees.

**Figure 4:**
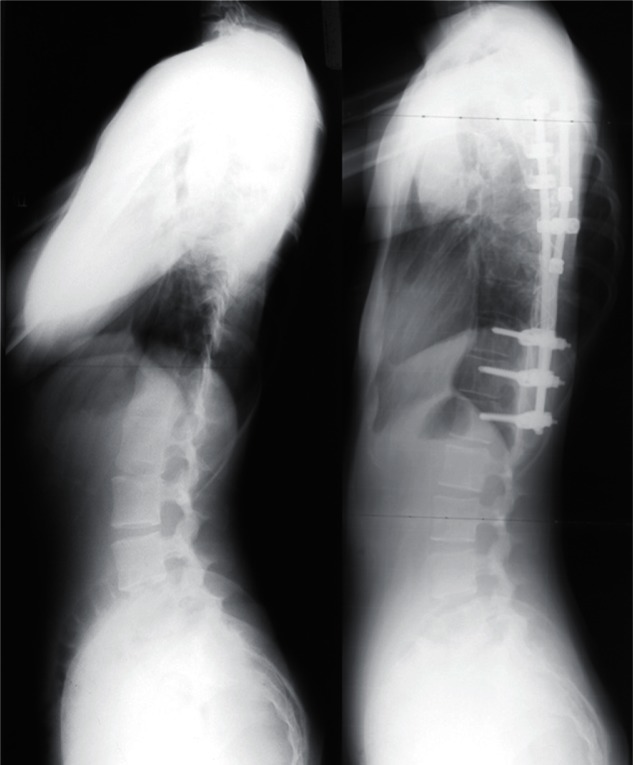
Hybrid constructs using pedicle screws, hooks, and wires. Lateral radiographs before and after surgery.

Posterior fusion with instrumentation has been the standard form of surgical treatment for scoliosis. The first instrumentation system of the modern era was introduced by Paul Harrington.^7^ In his system, correction force was applied with distraction along the concavity of the curve. In the second generation instrumentation system developed by Cotrel and Dubousset,^8^ on which all the current systems are modeled, correction was attempted by the rod-rotation maneuver. Segmental spinal instrumentation developed by Luque^9^ has been widely used mainly for neuromuscular scoliosis. In modern instrumentation systems, more anchors are used to connect the rod and the spine, resulting in better correction and less frequent implant failures.^10^ Use of segmental pedicle screw constructs (Figs. 1, 2) or hybrid constructs using pedicle screws, hooks, and wires (Figs. 3, 4) are the trend currently.

By the segmental pedicle screw concept, the idiopathic thoracic curves of an average of 51 degrees were corrected to 16 degrees (69% correction) with a follow-up for a minimum of five years.^11^ Although 1.5% of the screws inserted in the thoracic level were malpositioned, they did not cause neurologic complications or adversely affect the long-term results. Using hybrid constructs with hooks, apical sublaminar wires, and pedicle screws, the correction rate was 63% with a follow-up for a minimum of five years.^12^ No difference was found between the apical sublaminar wires and pedicle screws for initial correction (67.4% vs. 68.1%), loss of correction (4.6% vs. 5.1%), operating time (350 minutes vs. 357 minutes), and satisfaction of the patients, but intraoperative blood loss was more with wires (1791 ml vs. 824 ml) and instrumentation cost was higher with screws (8341 USD vs. 13462 USD).^13^ Another concern with segmental pedicle screw constructs is that vigorous correction of a major curve is an overcorrection, relative to the flexibility of the upper compensatory curve.^14^ Generally, the extent of fusion level is determined by the flexibility of the curves demonstrated on the radiographs taken in positions such as supine side bending, fulcrum side bending, traction, or push-prone position.^15^–^17^ With segmental pedicle screw technique, to avoid the postoperative shoulder imbalance, fusion has to be extended frequently to the upper thoracic vertebrae, which is not included in the fusion with other techniques.

## Anterior Instrumentation

**Figure 5:**
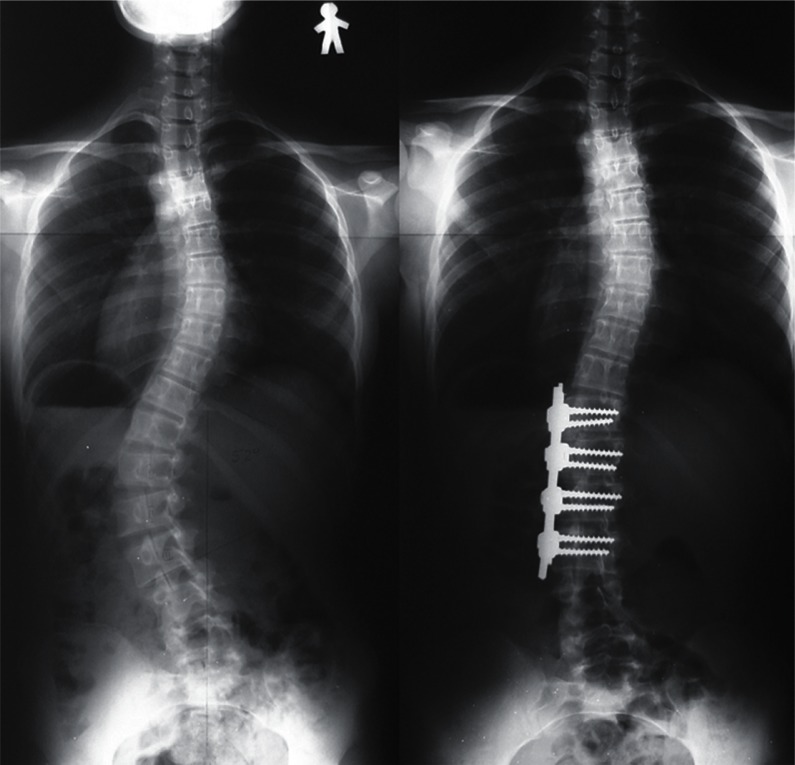
Anterior instrumentation surgery. Left thoracolumbar curve between the T11 and L4 was corrected from 52 to 19 degrees (By courtesy of Dr. Tomasz Kotwicki).

**Figure 6:**
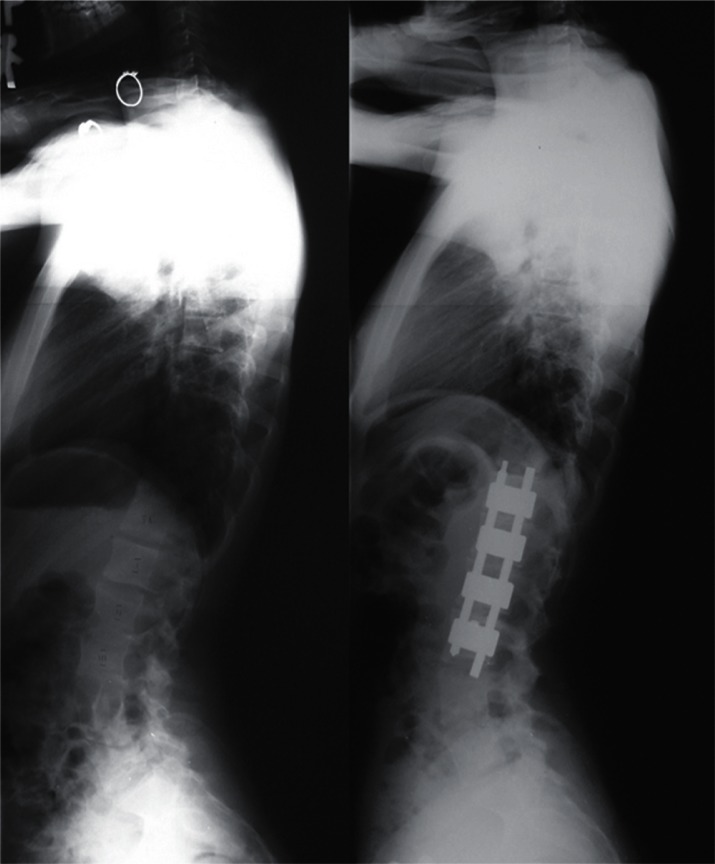
Anterior instrumentation surgery. Lateral radiographs before and after surgery (By courtesy of Dr. Tomasz Kotwicki).

Dwyer and Zielke^18^,^19^ were the pioneers of the anterior instrumentation surgery (Figs. 5, 6), which was the choice of treatment for thoracolumbar and lumbar scoliosis because better correction could be obtained with shorter fusion levels. Moreover, anterior instrumentation for the thoracic curve using video assisted thoracoscopy was developed.^20^ Initial enthusiasm for this surgery due to expectations of decreased postoperative pain and patients’ satisfaction with less operative scar has faded out because the thoracic aorta is at risk if the screw penetrates the cortex on the opposite side,^21^,^22^ and disruption of the chest cage during the surgical treatment affects pulmonary function after surgery.^23^ Thoracic curve can be treated successfully with posterior instrumentation surgery without affecting pulmonary function. For the treatment of single thoracic curve, posterior fusion group demonstrated greater curve correction (62% versus 52%) and greater rib hump correction (51% versus 26%) than the anterior fusion group.^24^ Recently, superiority of anterior instrumentation surgery for the thoracolumbar and lumbar scoliosis has been questioned as well. In adolescent idiopathic thoracolumbar and lumbar scoliosis, the coronal correction with a minimum of a 2-year follow-up was compatible between the posterior segmental pedicle screw instrumentation group and anterior instrumentation group (68% vs. 67%), but the duration of surgery was significantly shorter (189 minutes vs. 272 minutes) as well as the length of hospital stay (6.2 days vs. 8 days), in the posterior segmental pedicle screw group than in the anterior instrumentation group.^25^

## Osteotomy in Combination with Instrumentation

Various osteotomies are conducted in combination with instrumentation. These osteotomies are rarely indicated in the primary surgery of idiopathic scoliosis. For the treatment of relatively mild kyphotic deformities such as Scheuermann’s kyphosis, total facet joint resection, which is called as Smith-Peterson osteotomy,^26^ or Ponte osteotomy^27^ are performed. To treat more rigid, local or focal kyphotic deformity, pedicle subtraction osteotomy^28^ is performed. For severe deformity with limited flexibility including revision surgeries for the previous failed fusion surgery, vertebral column resection^29^ is sometimes conducted. This is one of the most challenging procedures among the treatment of the spinal deformities.

## Fusionless Surgery

Various attempts are being made using fusionless surgery to control growth, to avoid fusion, to delay the timing of the definitive fusion surgery, and to increase the volume of the thorax.

## To Control Growth

Epiphysiodesis on the convex side of the deformity with or without instrumentation is a technique that provides gradual progressive correction and arrest of the progression of curves. Some authors found that the arrest of anterior and posterior growth alone are not effective in preventing the progression of deformity in infantile scoliosis.^30^ On the contrary, others showed that stapling the anterior vertebral spinal growth plates could control worsening of the curve in patients with adolescent idiopathic scoliosis.^31^ By using newly designed biocompatible shape memory metal alloy staples, 6 of 10 patients with average curve magnitude of 35 degrees were stabilized during the follow-up period which was more than 1-year. To avoid overtreatment of a relatively small, non-progressive curve with this technique, definite and solid criteria for hallmarking a curve as non-progressive should be established first.

## To Avoid Fusion

By fusion surgery, segmental motion of the vertebral column is eliminated. To avoid fusion in patients with paralysis, for whom maintaining spinal flexibility and mobility is more desirable, fusionless, vertebral wedge ostetomies are developed for the treatment of progressive paralytic scoliosis of skeletally immature children with spinal cord injury or myelodysplasia.^32^ A specially designed implant system is used to assist with correction and maintenance of alignment. Twelve weeks following the initial surgery, a second surgery is necessary to remove parts of the implants. This technique may be used for idiopathic scoliosis in future.

**Figure 7:**
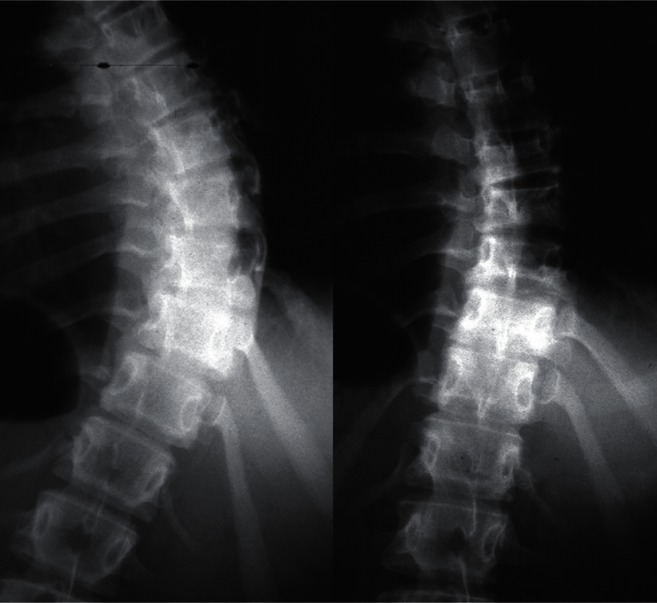
Multiple vertebral wedge osteotomy. Right thoracic curve between the T5 and T12 corrected from 56 to 26 degrees.

**Figure 8:**
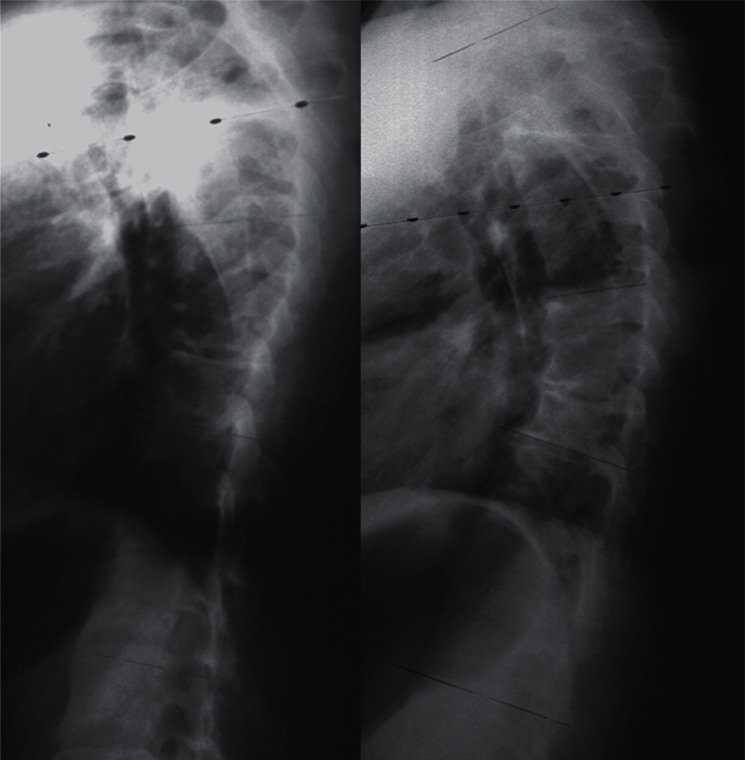
Multiple vertebral wedge osteotomy. Lateral radiographs before and after surgery.

For right thoracic curve with idiopathic scoliosis, multiple vertebral wedge osteotomies without fusion (Figs. 7, 8) are performed.^33^ Twenty patients were treated with osteotomies on an average of 3.6 periapical vertebrae and followed-up for 8.9 years on an average. There were no neurologic complications. For four patients with Risser 0 or I, the average curve magnitude was 74.8 degrees before surgery and 67.5 degrees at the latest follow-up (correction rate was 9.8%), whereas, for 16 patients with Risser IV or V, the curve was 61.3 degrees before surgery and 43.3 degrees at the latest follow-up (correction rate 29.4%).

## Delay of the Timing of Fusion

Fusion surgery at a very young age results in short trunk relative to the extremities. It also affects the development of the lung. To provide correction and maintain it during the growing years, while allowing spinal growth, in patients with early onset scoliosis, technique of instrumentation without fusion or with limited fusion using Harrington rod, Cotrel-Dubousset rod, or Luque rod were developed.^34^,^35^ Recently, the technique using Isola dual rod instrumentation has been developed.^36^ Upper and lower foundations are made bilaterally using hooks or pedicle screws as anchoring devices. Each foundation is connected to a rod, and the rods are connected by a tandem connector, which is placed at the thoracolumbar junction on each side. Lengthening is performed usually every 6 months by distraction inside the tandem connector or between the rod and the tandem connector. Once maximum spinal growth is accomplished, definitive final arthrodesis with instrumentation is performed. Between 1993 and 2001, 23 patients with various etiologies underwent this treatment procedure at an average age of 5.4 years. The average curve magnitude was 82 degrees before surgery, 38 degrees after the initial surgery, and 36 degrees after an average of 6.6 times of lengthening procedures. The length of thoracic and lumbar spine increased by 5 cm at the initial surgery and 4.7 cm in addition, during the lengthening period.

## To Increase the Volume of the Thorax

To treat thoracic insufficiency syndrome associated with fused ribs and congenital scoliosis, vertical expandable prosthetic titanium ribs (VEPTR) have been developed.^37^ After an opening-wedge thoracostomy, the acute correction is stabilized by VEPTR. The device is extended from the cephalad rib to the caudal rib, to the lumbar spine, or to the posterior iliac crest. Following the initial implantation, the devices are expanded at scheduled intervals of four to six months. Twenty-seven patients underwent this procedure at an average age of 3.2 years and were followed-up for 5.7 years. Vital capacity increased significantly; moreover, the deformity due to scoliosis was indirectly corrected from 74 to 49 degrees at the last follow-up.

## Conclusions

The indications for surgical treatment of scoliosis, results of the innovative surgical techniques, in terms of, posterior fusion with instrumentation, anterior fusion with instrumentation, and various kinds of fusionless surgery are discussed.
